# Scorpion Venom Antimicrobial Peptides Induce Caspase-1 Dependant Pyroptotic Cell Death

**DOI:** 10.3389/fphar.2021.788874

**Published:** 2022-01-10

**Authors:** Ranwa A. Elrayess, Mahmoud E. Mohallal, Yomn M. Mobarak, Hala M. Ebaid, Sarah Haywood-Small, Keith Miller, Peter N. Strong, Mohamed A. Abdel-Rahman

**Affiliations:** ^1^ Biomolecular Sciences Research Centre, Sheffield Hallam University, Sheffield, United Kingdom; ^2^ Zoology Department, Faculty of Science, Suez Canal University, Ismailia, Egypt; ^3^ Zoology Department, Faculty of Science, Suez University, Suez, Egypt

**Keywords:** Antimicrobial peptides, scorpio maurus palmatus, Smp24, Smp43, cell death, pyroptosis, caspase-1, IL-1b

## Abstract

Within the last decade, several peptides have been identified according to their ability to inhibit the growth of microbial pathogens. These antimicrobial peptides (AMPs) are a part of the innate immune system of all living organisms. Many studies on their effects on prokaryotic microorganisms have been reported; some of these peptides have cytotoxic properties although the molecular mechanisms underlying their activity on eukaryotic cells remain poorly understood. Smp24 and Smp43 are novel cationic AMPs which were identified from the venom of the Egyptian scorpion *Scorpio maurus palmatus*. Smp24 and Smp43 showed potent activity against both Gram-positive and Gram-negative bacteria as well as fungi. Here we describe cytotoxicity of these peptides towards two acute leukaemia cell lines (myeloid (KG1-a) and lymphoid (CCRF-CEM) leukaemia cell lines) and three non-tumour cell lines CD34^+^ (hematopoietic stem progenitor from cord blood), HRECs (human renal epithelial cells) and HaCaT (human skin keratinocytes). Smp24 and Smp43 (4–256 µg/ml) decreased the viability of all cell lines, although HaCaT cells were markedly less sensitive. With the exception HaCaT cells, the caspase-1 gene was uniquely up-regulated in all cell lines studied. However, all cell lines showed an increase in downstream interleukin-1*β* (IL-1*β*) expression. Transmission electron microscope studies revealed the formation of cell membrane blebs and the appearance of autolysosomes and lipid droplets in all cell lines; KG1-a leukemia cells also showed the unique appearance of glycogen deposits. Our results reveal a novel mechanism of action for scorpion venom AMPs, activating a cascade of events leading to cell death through a programmed pyroptotic mechanism.

## Introduction

Antimicrobial peptides (AMPs) are found throughout the animal kingdom and represent an ancient host defence mechanism of the innate immune system (Zasloff, 2002; Lai and Gallo, 2009; Bahar and Ren, 2013). They are increasingly recognized as a potential source of new broad-spectrum antibiotics, to combat the steady and alarming rise in the resistance of pathogenic microorganisms to conventional drugs (Guilhelmelli et al., 2013; Nuti et al., 2017). AMPs have also been shown to selectively target several types of tumour cells, with the prospect of being able to develop these peptides as novel anti-cancer drugs (Deslouches and Di 2017).

The attractiveness of AMPs in antimicrobial therapy lies in their mechanism of action as membrane disruptive agents, against which microbes have little natural resistance. AMPs are predominantly cationic, amphipathic molecules. Their selectivity arises from a primary electrostatic interaction of their charged surface with negatively charged prokaryotic membranes (Huang, 2000; Zasloff, 2002). Although healthy eukaryotic cells have a zwitterionic external membrane, cancer cells are characterized by the transbilayer movement of phosphatidylserine to the outer membrane leaflet, thereby endowing tumour cell membranes, analogously to prokaryotic cell membranes, with a negative charge (for a review, see Sharma and Kanwar, 2018). The appearance of phosphatidylserine on the outer membrane leaflet regulates malignant transformation by primarily suppressing anti-tumour immune responses (Utsugi et al., 1991; Fadok et al., 2000).

Clearly, the development of AMPs as anti-cancer agents (Schweizer, 2009; Wang and Wang, 2016; Crusca et al., 2018; Lei et al., 2019), will depend on developing selectivity by creating a sufficient distinction (effective therapeutic index) between their cytotoxic effects on target tumour cell membranes with respect to the membranes of normal cells. Understanding the cytotoxic mechanism by which AMPs exert their action is therefore crucial to the development of these potentially very attractive agents, as therapeutic drugs.

Abdel-Rahman et al. (2013) identified two novel amphipathic cationic AMPs (Smp43 and Smp24) through cDNA sequencing of the venom gland of the Egyptian scorpion *Scorpio maurus palmatus*. Both peptides showed a potent activity against both Gram-positive and Gram-negative bacteria as well as fungi. Both peptides formed pores in model prokaryotic and eukaryotic phospholipid membranes, although their detailed mechanism of action differed and the same peptide was shown to act by different mechanisms according to the cell membrane composition of the host cell (Harrison et al., 2016b; Heath et al., 2018). Both peptides had cytotoxic effects on various cancer cell lines (liver, myeloid and lymphoid leukaemia). In contrast, keratinocytes were markedly less sensitive and although Smp24 was cytotoxic to erythrocytes, Smp 43 was non-haemolytic at all concentrations tested (Harrison et al., 2016b; Elrayess et al., 2019).

Cell death can occur in both a controlled and uncontrolled manner (for a recent review, see Darcy, 2019). The loss of cell membrane integrity has conventionally been associated with the process of necrosis, an uncontrolled form of cell death induced by external injury such as hypoxia or inflammation which often involves the up-regulation of pro-inflammatory molecules, resulting in rupture of the cell membrane. In contrast, apoptosis is a programmed, tightly controlled form of cell death that crucially, does not involve damage to the cell membrane. It is now recognised that, in conjunction with cell death programmes that do not disrupt cell membranes, some forms of necrosis are not passive and can also be programmed by the cell in a process known as pyroptosis (Hersh et al., 1999; Bergsbaken et al., 2009; Frank and Vince, 2019). Pyroptosis is a lytic, inflammatory type of necrotic cell death, characterized by cell swelling and the release of inflammatory factors. It is highly regulated. The expression of caspase-1 is a primary marker of pyroptosis and results from the activation of the pro-enzyme by pattern recognition receptors of inflammasome complexes. Gastrodermin-D (GSDMT) is a key substrate of caspase-1 and the enzyme catalyses the release of an N-terminal fragment (GSDMT-cNT) which causes cells to swell until they rupture. Caspase-1 also processes inflammatory cytokines such as interleukin-1*β* (IL-1*β*) and interleukin-18, which can be released by channels formed by GSDMD-cNT. Both the activation of caspase-1 and the increased expression of IL-1*β* are key pyroptotic markers, in clear distinction from activation by caspases 3/7 and 8/9, which are hallmarks of apoptosis.

Pyroptosis is increasingly recognised to play an important role in cancer (for recent reviews, see Xia et al., 2019; Fang et al., 2020). Intriguingly, pyroptosis may play dual but opposite roles in both promoting and inhibiting tumorigenesis. On the one hand, the many inflammatory mediators released in pyroptosis are closely related to tumorigenesis and the resistance to chemotherapeutic drugs. In contrast, triggering the pyroptosis of tumour cells can offer a novel way of inhibiting tumour cell expression.

Some AMPs can exploit both differences in membrane charge and cell stability to specifically target certain cancer cells (Rathinakumar et al., 2009; Mahlapuu et al., 2016; Wang et al., 2017), suggesting that these peptides are potential new anti-cancer agents and encouraging further development of other classes of AMPs in this area. When examining the effects of Smp24 and Smp43 in morphological studies (Elrayess et al., 2019), we found suggestive evidence of pyroptosis. Here we have studied these preliminary observations in greater detail and suggest that Smp24 and Smp43 exert pyroptotic effects in both cancer and non-cancer cells, with the release of inflammatory cytokines. The pyroptotic effects in cancer cells are mediated by the activation of caspase-1, whereas the release of IL-1*β* in normal cells appears to be both dependent and independent of caspase-1.

## Materials and Methods

### Peptides and Materials

Smp24 (IWSFLIKAATKLLPSLFGGGKKDS) and Smp43 (GVWDWIKKTAGKIWNSEPVKALKSQALNAAKNFVAEKIGATPS) were synthesized (>90% pure) using solid-phase chemistry and were purchased from Think Peptides (Oxford, United Kingdom). DMEM media was obtained from Lonza (Cologne, Germany). Epithelial growth media was obtained from Innoprot (Deria, Spain). 96-well white microplates were obtained from Fisher Scientific (Loughborough United Kingdom). PCR primers were obtained from Applied Bioscience (Warrington, United Kingdom). Interleukin 1B antibody was obtained from Santa Cruz (Heidelberg, Germany). Fluorescent IRDye 800 CW goat anti-rabbit secondary antibody was obtained from LICOR (Cambridge, United Kingdom). Caspase activity (caspases 3/7,8 and 9) was measured using a Caspase-Glo® Assay kit obtained from Promega (Southampton, United Kingdom). Caspase-1 inhibitor (Z-WEHD-FMK) was obtained from R&D systems (Abingdon, United Kingdom). All other reagents were the highest grade available and were obtained from Sigma (Gillingham, United Kingdom).

### Cell Lines and Culture Conditions

Non-tumour haematopoietic progenitor stem cells (CD34^+^) were obtained from Stem Cell Technologies (Grenoble, France). Primary human renal epithelial cells (HRECS) were obtained from Innoprot. Immortalized human skin keratinocytes (HaCaT) were obtained from Cell Lines Service (Eppelheim, Germany). Human lymphoid leukaemia cells (CCRF-CEM, acute lymphoblastic leukaemia, ATCC: CCL-119) and human myeloid leukaemia cells (KG1-a, acute mylogenous leukaemia, ATCC: CCL-243) were obtained from the ATCC (Teddington, United Kingdom). MycoAlert^TM^ *mycoplasma* detection kits were obtained from Lonza (Cologne, Germany).

Suspended cells (CD34^+^, CCRF-CEM and KG1-a) were seeded in T75 cm^2^ flasks in RMPI 1640 medium supplemented with 10% (v/v) foetal bovine serum (FBS), 1.5 mM L-glutamine and 100 µg/ml penicillin/streptomycin. HaCaT cells were seeded in T75 cm^2^ flasks in DMEM medium supplemented with 10% FBS, 1.5 mM L-glutamine and 100 µg/ml penicillin/streptomycin. HRECs were seeded in T75 cm^2^ flasks in epithelial cell media containing 2% FBS, 1% epithelial cell growth supplement (EPICGS) and 1% penicillin/streptomycin. Cells were incubated at 37°C with 5% CO_2_ and tested regularly for *mycoplasma* contamination; all cells were negative throughout the study.

### Hoechst 33342 and Propidium Iodide (PI) Staining

Nuclear morphology was assessed by fluorescence microscopy following Hoechst 33342 and propidium iodide (PI) double staining. Cells were seeded in 96 well plates (2.5 × 10^4^ cells/well) and treated with peptides at different concentrations. Triton X-100 (10%) was used as positive control for necrosis while etoposide was used as positive control for apoptosis. Water was used as a negative control. Following treatments, double stain (10 µL) was added to all samples which were then incubated in the dark (30 min) and examined using a fluorescence microscope (Olympus BX60, Japan).

### Transmission Electron Microscopy

Cells were seeded in 6 well plates at 0.5 × 10^6^ cells/well and treated with different concentrations of Smp24 or Smp43 and water as vehicle control for 24 h. The treated cells were then harvested and centrifuged at 5000 RPM at 4°C (5 min). The supernatant was removed, and the cells were washed twice in 100 µL cold 0.1M PBS. The cells were fixed by adding 100 µL of 3% Glutaraldehyde in 0.1 M Sodium Phosphate Buffer, PBS (3 h at RT). The cells were then washed twice with cold 0.1M PBS each for 15 min. Then, they were fixed again by 1% aqueous osmium tetraoxide for 1 h. The cells were washed twice with 0.1 M PBS followed by ascending series of ethanol for dehydration (75%, 95%, and then twice in 100% each step 15 min). Cells were then placed in propylene oxide (two changes, 15 min each). Infiltration was accomplished by placing the cells in propylene oxide/Araldite resin (1:1) overnight at RT on a rotating mixer. Cells were then left in full strength Araldite resin (6 h, RT on a rotating mixer), after which they were embedded in fresh Araldite resin (48–72 h at 60°C). Semi-thin 0.5 μm sections were cut (Reichert Ultracut E ultramicrotome) and stained with 1% toludine blue in 1% borax. Ultrathin sections (70–90 nm thick) were similarly cut but stained with 3% aqueous uranyl acetate (25 min) followed by Reynold’s lead citrate (5 min). Sections were examined using a FEI Tecnai Transmission Electron Microscope (Gothenburg, Sweden) at an accelerating voltage of 80 Kv. Electron micrographs were taken using a Gatan digital camera (Abingdon, United Kingdom).

### Reverse Transcription Polymerase Chain Reaction

Reverse transcription polymerase chain reaction (qRT-PCR) analysis was used to analyze the gene expression of, *Casp1*, *Casp8*, *Capn5*, *MLKL*, *NLRP3*, and *GAPDH* (housekeeping gene) on cells treated with either Smp24 or Smp43. Water was used as a vehicle control. Full gene names, functions and primer details are shown in Supplementary Table S1.

### Caspase Activity

Cells were seeded into white 96-well plates at 2.5 × 10^4^ cells/well and treated with different concentrations of Smp24 and Smp43 (37°C, 24 h). Following treatments, 100 µL of Caspase-Glo^®^ (8, 9, 3/7) reagent was added to the cells, which were incubated (RT, 1 h) after initial mixing (1–2 min using a plate shaker at 300–500 rpm). Luminescence was measured using a Wallac Victor 2 1420 detector (Marshall Scientific, United Kingdom). Experiments were performed in triplicate in three independent repeats.

### Detection of Interleukin-1*β* (IL-1*β*) by Dot Blotting

Media from cell lines, treated with either Smp24 or Smp43 for 24 h (at respective LC_50_ concentrations, Elrayess et al., 2019) were concentrated and stored at −80°C until used. Media (2 µL) were spotted on the middle of nitrocellulose membranes placed in individual wells of a 6-well plate and left to dry. 1 ml blocking solution (TBS-T) (0.15M NaCl, 0.05% Tween-20, 25 mM Tris pH 7.5) was added and the plate incubated (1 h, RT with shaking). After removing the blocking solution, IL-1*β* rabbit antibody (500 µL, 1:200 diluted in blocking solution) was added to each well and incubated overnight (4°C with shaking). After washing the membranes (TBS-T, RT, 10 min × 3 times with shaking), goat anti-rabbit secondary antibody was added (1:10000 dilution) and incubated (1 h, RT with shaking in the dark). Membranes were finally washed (TBS-T, RT, 10 min with shaking x3) before being scanned. The experiment was performed in duplicate in three independent repeats.

### Immunocytochemical Detection of IL-1*β*

Adherent cells were seeded on cover slips (6 well plates, 5 × 10^5^ cells/well). Plates coated with poly-Lysine were used for suspended cells. Cells were treated (at respective LC_50_ concentrations for 24 h, with or without a caspase-1 inhibitor (Z-WEHD-FMK) before incubation with either Smp24 or Smp43 for 24 h at their respective LC_50_ concentrations (Elrayess et al., 2019). Cover slips were then washed (PBS x2), fixed (4% paraformaldehyde, 15min, RT) and washed again (PBS x2). Cells were permeabilized (100% ice cold methanol/acetone (1:1), 15 min, RT), blocked (5% bovine serum albumin in TBS-T, 1 h, RT with shaking) and incubated overnight with IL-1B antibody (1 µL/ml, 4°C with shaking). Cells were washed (TBS-T, RT, 10 min x 3 with shaking) and then incubated in the dark with Alexa fluor-488 goat anti-rabbit IgG (1 µL/ml, RT, 1 h with shaking). Cover slips were finally washed (TBS-T, 10 min, RT x3 with shaking) before mounting onto a glass slide in the presence of DAPI. Slides were examined using a fluorescence microscope (Olympus, BX60, United Kingdom) and images were captured (Micropublisher 5.0 RTV). Experiments were performed in triplicate with three independent repeats. Nigericin was used as positive control for maximum IL-1*β* release.

### Statistical Analysis

Data were analysed (unpaired student-*t* test) using Prism 6 software (Graph Pad). Means and standard errors were calculated and results were considered statistically significant when *p* ≤ 0.05 and highly significant when at *p* ≤ 0.001.

## Results

**FIGURE 1:**
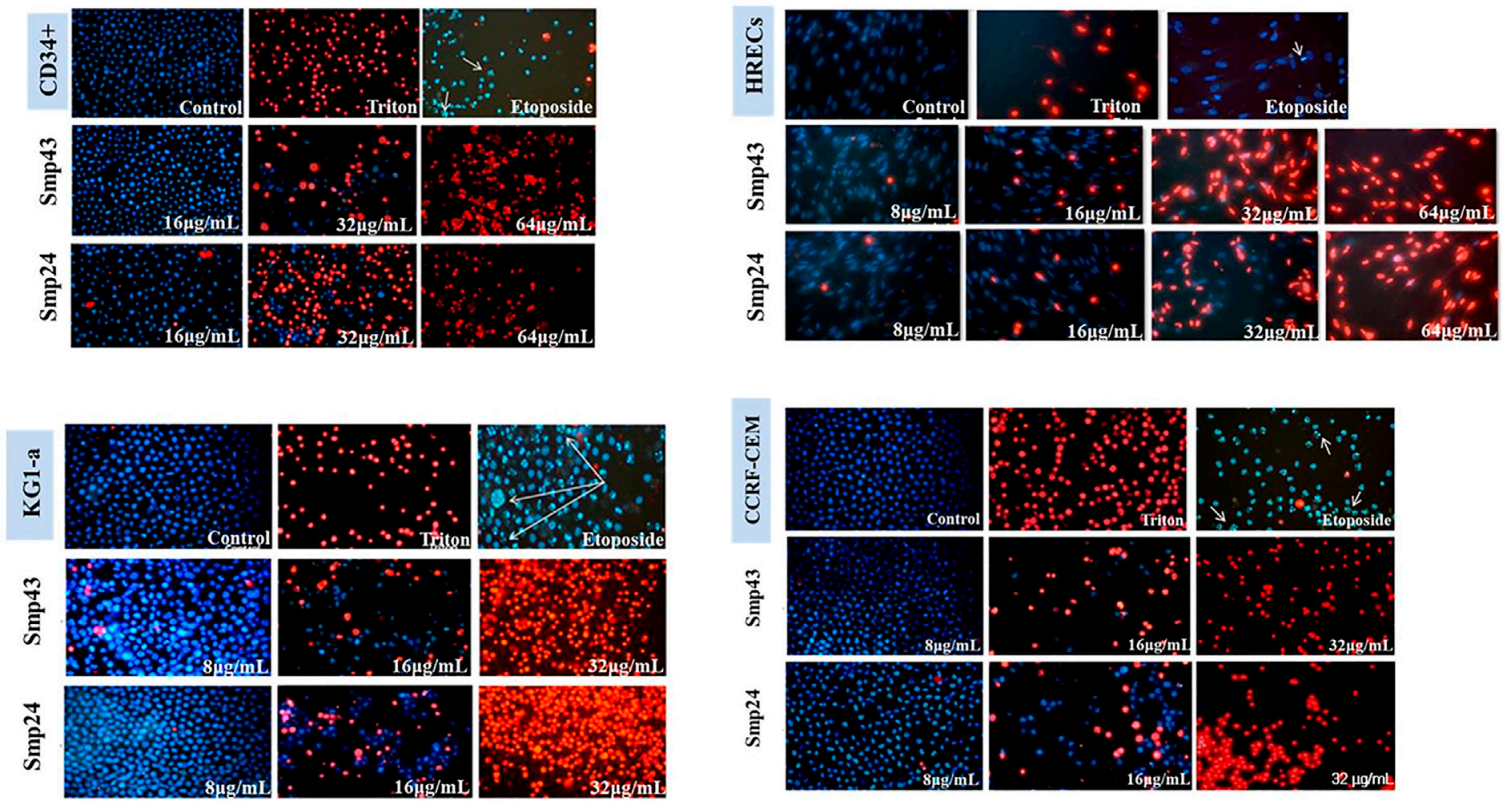
Changes in nuclear morphology of the non-tumour (CD34^+^ and HRECs) and tumour (KG1-a and CCRF-CEM) cell lines treated with different concentrations of Smp24 or Smp43 (8–256 μg/ml) showed significant increase in PI stain uptake in a concentration dependant manner (20X).

Previous results have demonstrated that Smp24 and Smp43 disrupted the integrity of cell membranes as evidenced by the release of cytoplasmic lactate dehydrogenase in a range of tumour and non-tumour cell lines as well as primary cells^**18**^. To examine this cytotoxic event in more detail, all cell lines were double stained with Hoechst 33342 dye and propidium iodide (PI) after treatment with either Smp24 or Smp43. All cell lines showed a concentration-dependent increase in PI uptake as evidenced by red-stained nuclei, as compared with controls which were stained blue with Hoechst 33342 dye (Figure 1; Supplementary Figure S1). Triton X-100 (10%) was used as positive control for necrotic cell death (100% PI uptake) and etoposide was used as positive control for apoptotic cell death. All treated cells had homogeneously dispersed chromatin, indistinguishable from controls and there was no evidence of highly condensed chromatin or small, dispersed apoptotic bodies, typical of an apoptotic process. These results suggest that the cytotoxic effects of Smp24 and Smp43 are due to a lytic mechanism.

**FIGURE 2:**
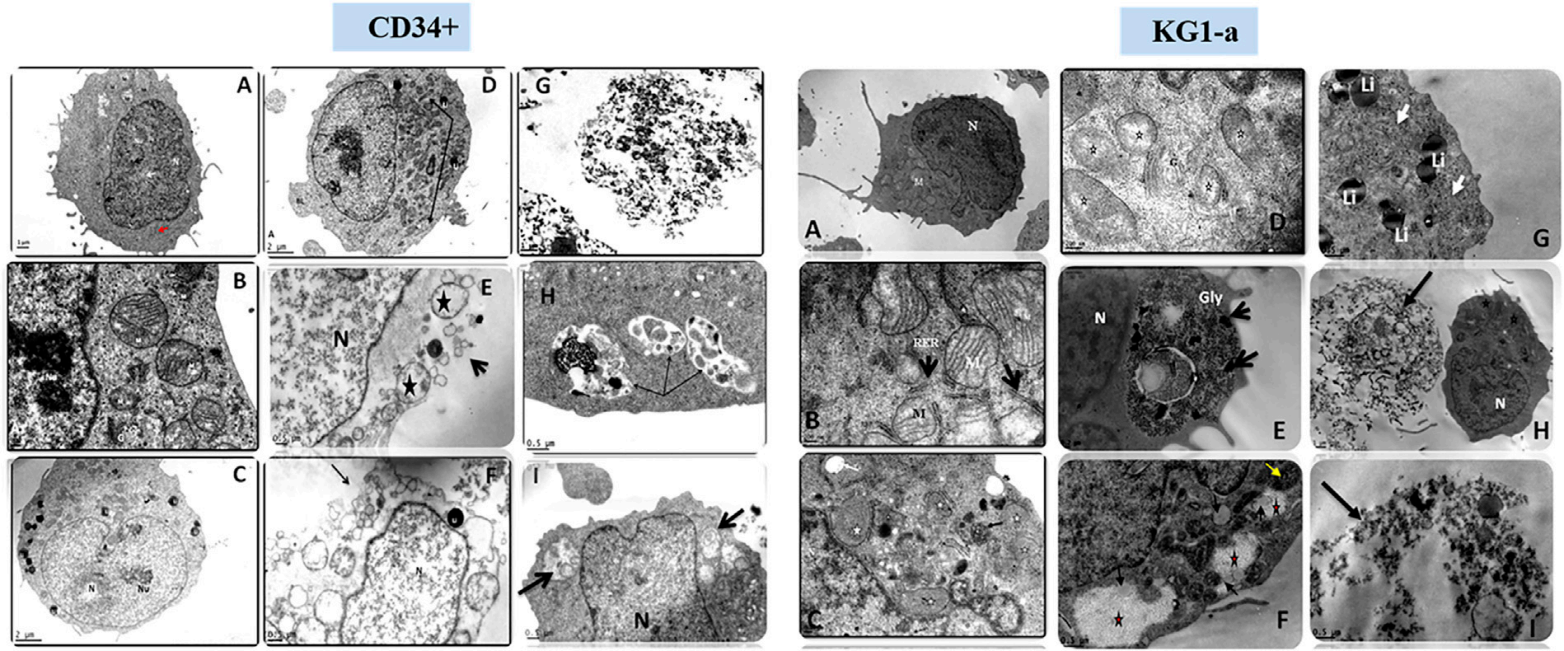
Transmission electron micrograph of CD34^+^ and KG1-a cells showing the effects of Smp24 and Smp43 treatment on their cell components. CD34^+^, **(A,B)**, control untreated cells showing normal ultrastructure of the cell. **(C,D)** cells treated with 1/2 LC_50_ concentration of Smp43 and Smp24, respectively, showing obvious increase in cell size, mitochondria were with inconspicuous cristae (star, arrow) and many lipid droplets (Li). **(E–I)** cells treated with LC_50_ concentration of Smp24 and Smp43. Most of cells were completely lysed **(G)** while other cells **(E,F)** showed ruptured cell membrane (arrow), karyolysis nucleus (N) and many lucent vacuoles (star). Autolysosomes were also noted (arrow) in the cytoplasm **(H,I)**. KG1-a, control untreated cells showing normal ultrastructure of the cell. **(C,D)**, cells treated with 1/2 LC_50_ concentration of Smp43 and Smp24, respectively, showing mitochondria were with inconspicuous cristae (star) and appearance of lucent vacuoles (White arrow) and multivesicular body (black arrow). **(E–I)** cells treated with LC_50_ concentration of Smp24 and Smp43. Most of cells were completely lysed **(H,I)** while other cells **(G)** showed deposition of lipid droplets (Li), and deposition of glycogen granules **(E,F)** inside lysosomes (Gly, star) deposits of dense pigments (arrow).

Scanning EM studies of cells treated with either Smp24 or Smp43 revealed the loss of cell membrane microvilli and filopodia. The presence of pores in many cell membranes was also evident (Elrayess et al., 2019). In addition, transmission EM studies identified the existence of vacuolized cytoplasm and the complete absence of intracellular organelles in many cells (Figure 2; Supplementary Figure S2). Compelling evidence of intracellular damage in other cells was widespread, e.g., inconspicuous mitochondrial cristae, the dilatation and fragmentation of cisternae in endoplasmic reticula and the shedding of microvesicles. The presence of autophagosomes, lipid droplets, myelinamellar structures and multivesicular bodies were seen in all cells examined. In summary, the demonstration of membrane blebbing producing apototic-like cell body protrusions, the formation of membrane pores and evidence of cell swelling prior to cell lysis, strongly suggests that Smp24 and Smp43 are causing a pyroptotic response in all the cells examined here.

**FIGURE 3:**
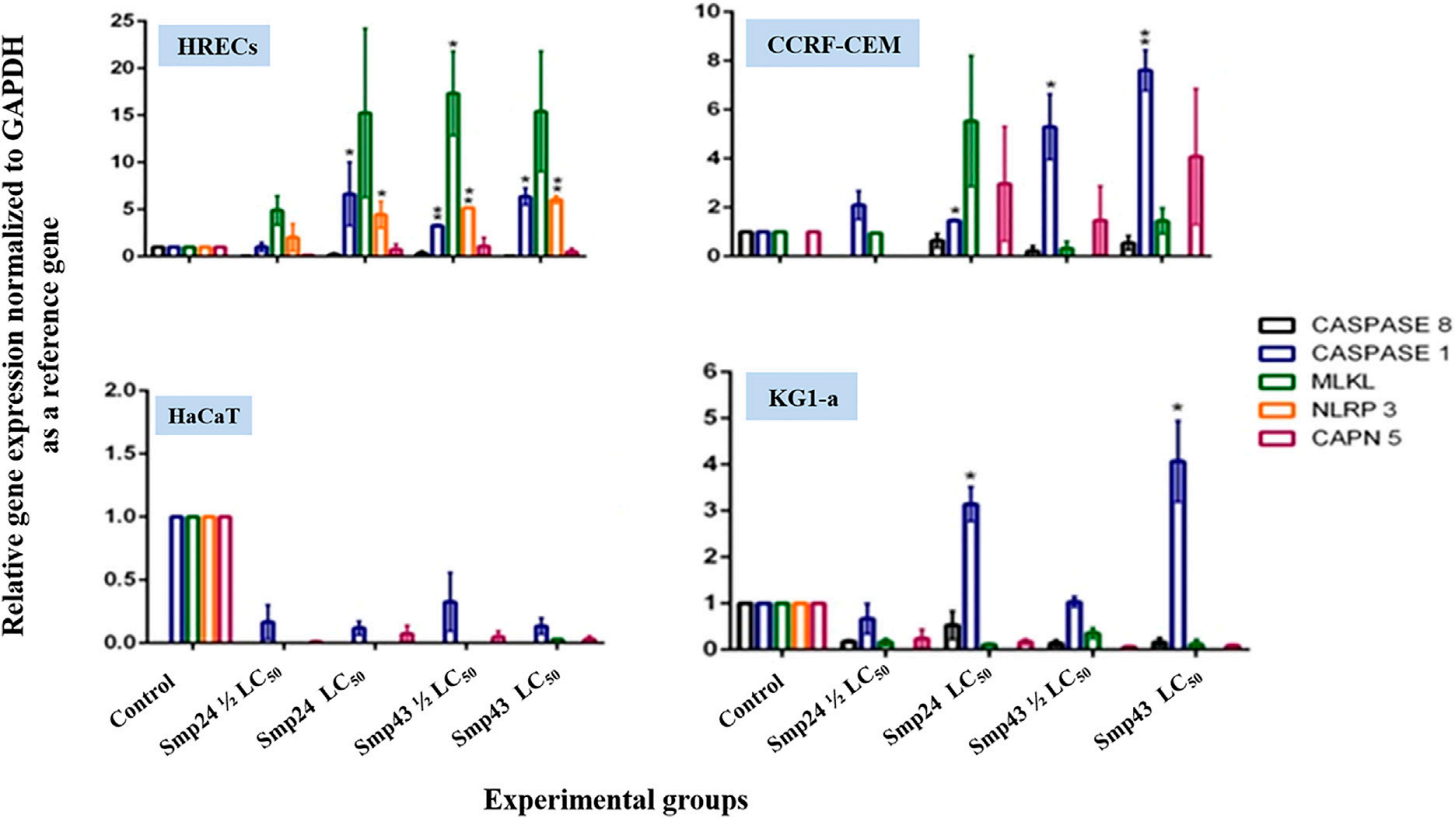
Charts showing relative genes expressions quantified by a qRT-PCR after treating non-tumour (HRECs and HaCaT) and tumour (CCRF-CEM and KG1-a) cell lines, with Smp24 and Smp43. All gene expressions were normalized to the expression of the housekeeping gene, GAPDH. The data represented as mean ± SE. The statistical significance (*p ≤ 0.05, **p ≤ 0.001) was determined by comparison with the control using student t-test.

The relative expression of various target genes after treating cells with either Smp24 or Smp43 (at concentrations ranging from 0.5-2 LC_50_) was examined by qRT-PCR analysis. The *Casp1* gene was significantly (*p* ≤ 0.05) up-regulated in all cells except primary epithelial cells (Figure 3). In contrast, *Casp8* was significantly down-regulated (*p* ≤ 0.05) across all cells studied. *NLRP3* also showed significant up-regulation (*p* ≤ 0.05) in non-tumour cells but not in the tumour cell lines. *MLKL* and *CAPN5* gene expression were unaltered.

**FIGURE 4:**
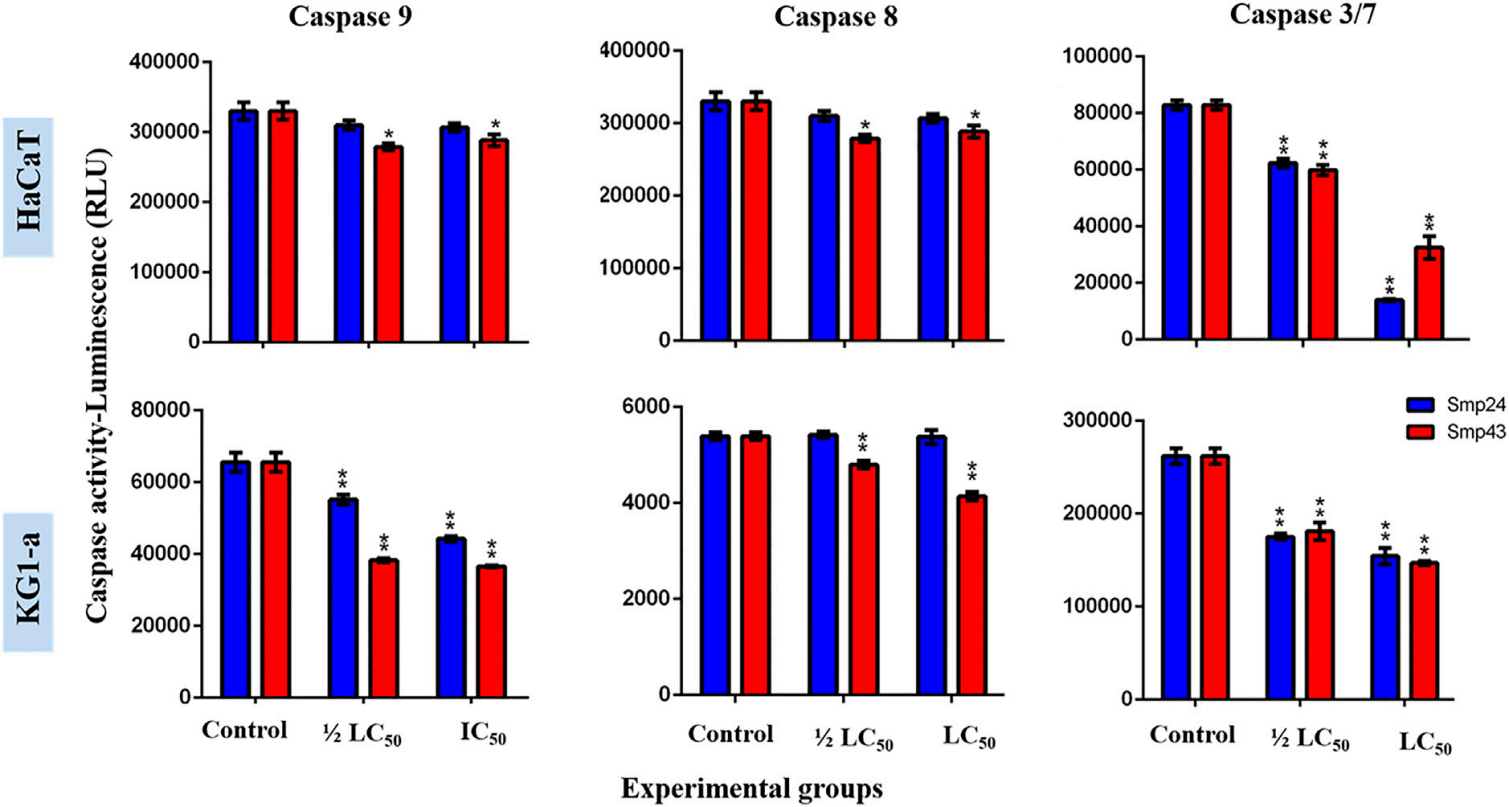
Charts showing the effect of Smp24 and Smp43 (1/2 LC_50_ and LC_50_) on the activity of caspase 8, 9 and 3/7 of representative non-tumour (HaCaT) and tumour (KG1-a) cell lines. The results showed no significant increase in caspases activities. The data represented as mean ± SE. The statistical significance (*p ≤ 0.05, **p ≤ 0.001) was determined by comparison with the control using student t-test.

At concentrations (1/2 LC_50_ and LC_50_) of either Smp24 or Smp43, no significant increase in either initiator caspase enzymatic activity (caspase-8 and 9) or executioner caspase enzymatic activity (caspase-3/7) was detected, as compared to their controls (*p* ≤ 0.05) (Figure 4; Supplementary Figure S3).

**FIGURE 5:**
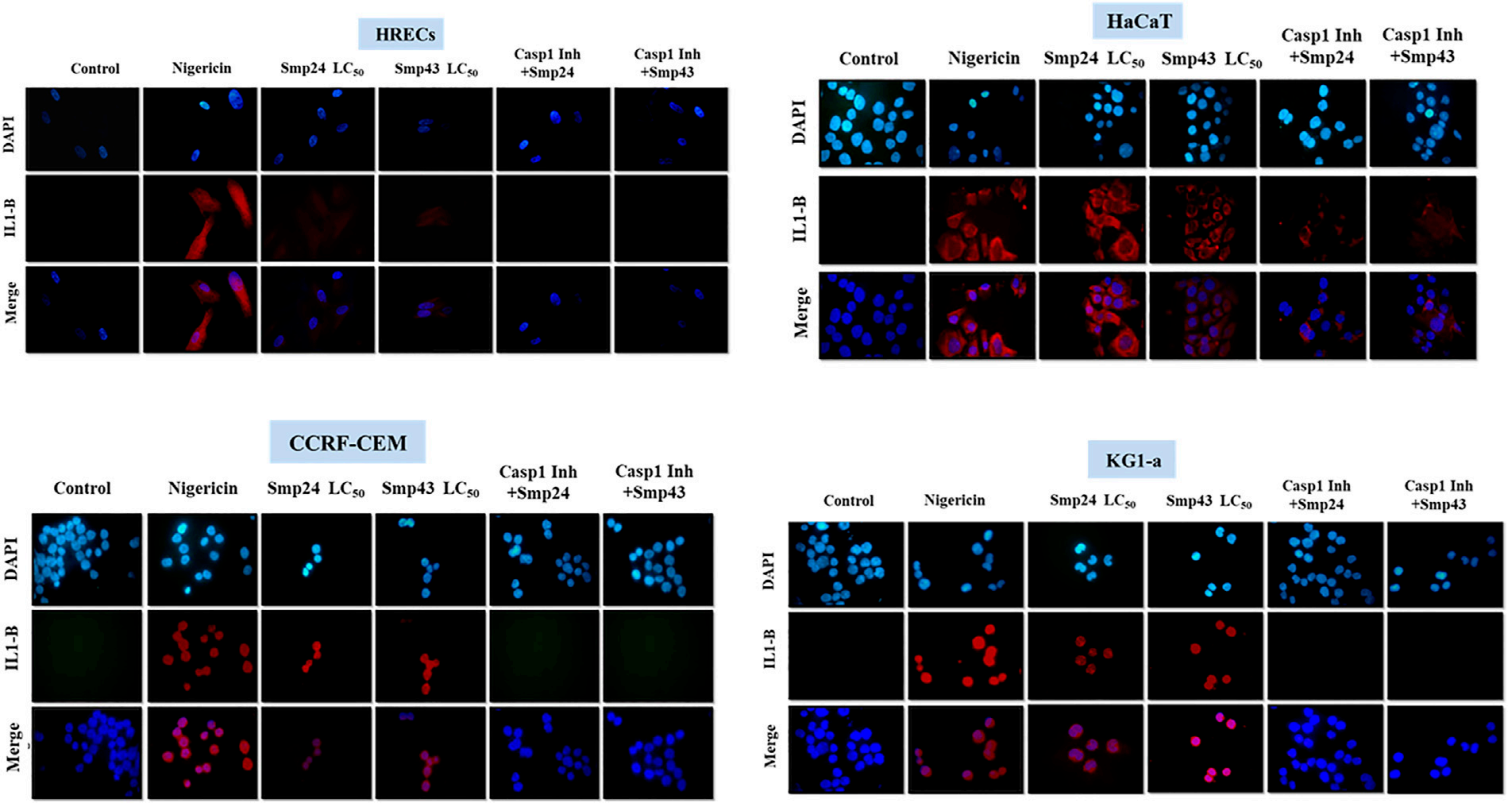
Intracellular IL-1B localization was visualized using fluorescence microscopy after treatment of non-tumour (HRECs and HaCaT) and tumour (CCRF-CEM and KG1-a) cell lines with Smp42 and Smp43 (LC_50_ concentrations). Cells were stained with antibody against IL-1B followed by Alexa Fluor 647 donkey anti-rabbit (red colour) and countered with a Nuclear DNA dye, DAPI (blue colour) (X50).

The pro-inflammatory cytokine IL-1*β* was detected by dot blot analysis in the culture supernatants of all cells treated with either Smp24 or Smp43 (Supplementary Figure S4). Nigericin was used as a positive control to induce the expression of IL-1*β*. Immunofluorescence microscopy showed that IL-1*β* was present in the cytoplasm of both tumour and non-tumour cells all cells treated with either Smp24 or Smp43 (Figure 5). IL-1*β* was absent from all cells pre-treated with the synthetic peptide caspase-1 inhibitor, Z-WEHD-FMK, with the exception of HaCaT cells, which still showed presence of the cytokine.

## Discussion

AMPs play an important role in the defence system of all organisms against diverse microbial infections. Smp24 and Smp43 are novel cationic AMPs characterized from the venom gland of the Egyptian scorpion *Scorpio maurus palmatus* (Abdel-Rahman et al., 2013). Both peptides showed potent antibacterial and antifungal activities (Harrison et al., 2016a,b), as well as cytotoxic effects in both tumour and non-tumour cell lines at higher concentrations (Elrayess et al., 2019), although tumour cells were markedly more sensitive to the effects of Smp peptides than either stem cells or primary epithelial cells. In previous studies we have shown that Smp24 may be favoured over Smp43 for potential development as an anti-microbial agent (Harrison et al., 2016a,b). In contrast, toxicity studies on leukemia cell lines have demonstrated that Smp43 may have higher potential as an anti-cancer agent (El-Rayess et al., 2019).

Although the classical pyroptosis pathway is caspase-1 dependent, non-classical pathways also exist that are independent of caspase-1 activation. With the exception of HaCaT cells, all cell lines, treated with either Smp24 or Smp43, revealed an up-regulation of *Casp1* and a down-regulation of *Casp8.* The Nod-like receptor, *NLRP3* was up-regulated in CD34^+^ and HRECs cell lines treated with both peptides, suggesting that they induce caspase-1-dependent pyroptotic cell death via an NLRP3 inflammasome complex. In contrast, leukaemia cell lines (CCRF-CEM and KG-1a) probably activate caspase-1 through different inflammasome complexes. Although morphological evidence suggests that HaCaT cells undergo pyroptotic death on treatment with both Smp24 and Smp43, these must do so by non-classical pathways.

Other AMPs induce pyroptosis by both classical and non-classical pathways. For example, the human a-defensin HNP-1 AMP induces pyroptosis *via* NLRP3 inflammasome activation of caspase-1 (Chen et al., 2014), whereas *Tityus serrulatus* scorpion venom induced pyroptosis by a caspase-1 independent pathway, probably through Toll-like receptors (Zoccal et al., 2016).

In support of a pyroptotic mechanism, pro-inflammatory cytokine IL-1*β* was detected (either extracellularly or in the cytoplasm) of all cells treated with either Smp24 or Smp43, independent of whether cytokine release was a consequence of caspase-1 activation or not. The role of caspase-1 in triggering the activation of IL-1*β* was confirmed by immunofluorescence, using a caspase-1 inhibitor. The mechanism by which Smp24 and Smp43 stimulate the expression of IL-1*β* in HaCaT cells is presently not understood, although Caspase-4 or the NF-κB signalling pathway are attractive candidates.

Cytochemical evidence from the uptake of propidium iodide into cells treated with either Smp24 or Smp43 suggests that these peptides induce membrane rupture and cause cell death in a necrotic manner. Similar results have been observed using other DNA-binding dyes; temporin, isolated from the skin secretions of the Chinese brown frog and sclopendrasin, isolated from centipede secretions, both stimulate ethidium bromide uptake into various cancer cell lines (Hsu et al., 2011; Wang et al., 2013; Chen et al., 2014; Lee et al., 2015). Experiments with propidium iodide also support our earlier studies demonstrating the cytotoxic effects of Smp24 and Smp43 through LDH release and the loss of intracellular ATP (Elrayess et al., 2019). Membrane disintegration was also directly observed in this earlier study in scanning electron micrographs, with observation of pore formation, cell swelling and the presence of autolysosomes, all strongly suggesting that Smp24 and Smp43 causing a pyroptotic, rather than an apoptotic response. Morphological evidence for various classes of AMPs causing pyroptotic cell death in diverse tumour cell lines has been provided by several groups (Lehmann et al., 2006; Suttmann et al., 2008; Chang et al., 2011; Wang et al., 2013; Lu et al., 2016).

The present transmission electron microscopy studies also suggest that ultrastructural changes induced by Smp24 and Smp43 are a consequence of pyroptosis. Many cells showed an obvious increase in cell size and appearance of blebs in the cell membrane, in conjunction with shedding of microvesicles from the cell membranes and the formation of multivesicular bodies. Bleb formation has been suggested to be due to the formation of caspase-1-dependent membrane pores, with the consequent disruption of cellular ionic gradients and an increase in osmotic pressure, leading to water influx and cell swelling (Fink and Cookson 2006; Bergsbaken et al., 2009). Bubble-like protrusions (pyroptotic bodies) have also been observed by others (Chen et al., 2016). The shedding of microvesicles is an attractive mechanism for the release of IL-1*β* after caspase-1 activation (MacKenzie et al., 2001), although multiple other mechanisms have also been proposed, for example the involvement of secretory lysosomes and secretory autophagy and autolysosomes/autophagosomes have also reported in the present study (Bianco et al., 2005; Qu et al., 2007; Monteleone et al., 2015).

The appearance of glycogen deposits was uniquely seen in KG1-a leukaemia cells. Glycogen accumulation may also be the result of caspase-1 activation since the enzyme also regulates glycolysis and lipid metabolism (Ghukasyan and Heikal, 2014). The deposition of lipid droplets in cells treated with either Smp24 or Smp43 suggests that lipid metabolism is impaired; caspase-1 activation has been demonstrated to inhibit the clearance of triglyceride (Kotas et al., 2013) and stimulate lipid biogenesis by activating sterol regulatory element binding proteins, which are key regulators of cellular lipid levels (Gurcel et al., 2006; Im et al., 2011).

The observed karyolysis in the nuclei of all cells treated with either Smp24 or Smp43 could be attributed to the activation of nuclear endonucleases in injured cells (Williams et al., 2000), as caspase-1 can also stimulate endonuclease activity (Fink and Cookson, 2006).

Although Smp24 and Smp43 both convincingly stimulate pyroptosis, there are subtle differences in their mechanism of action, dependent on the cell line studied. This should not be surprising as we have already shown that the two peptides cause membrane degradation (albeit in bacteria) in different ways (Harrison et al., 2016a,b; Heath et al., 2018). Smp24 and Smp43 both stimulate the release of the inflammatory mediator IL-1*β* in all cell lines tested (with the sole exception of HaCaT cells), by a caspase-1 dependent pathway. KG1-a leukemic cells are also unique in showing glycogen deposits.

The high metabolic turnover of proteins in a cancer cell causes developmental deficiencies in their cytoskeleton with respect to normal cells (Lei et al., 2019). Cancer cells are also associated with an anomalous high density of acidic, negatively charged phospholipids on their outer membrane leaflet, more akin to prokaryotic membranes. Although AMPs have been shown to act by a variety of diverse mechanisms, both within the membrane and in the cytoplasm and cell nucleus (Graf and Wilson, 2019; Haney et al., 2019; Zasloff, 2019), all AMPs, as positively charged amphipathic molecules are initially targeted to the cell membrane through electrostatic interactions. Some AMPs can exploit both differences in membrane charge and cell stability to specifically target certain cancer cells (Rathinakumar et al., 2009; Mahlapuu et al., 2016; Wang et al., 2017), suggesting that these peptides are potential new anti-cancer agents and encouraging the further development of other classes of AMPs in this area.

Can we identify any distinguishing features that separate the effects of Smp24 and Smp43 between cancer and non-cancer cell lines from this study? Non-cancerous keratinocytes (HaCaT cells) are markedly less sensitive to the peptides as well as uniquely stimulating the release of IL-1*β* by a caspase-1 independent mechanism. In contrast, the leukaemia cell line KG1-a was unique in creating glycogen deposits. Although this is the first step, clearly the mechanism by which the peptides initially trigger inflammasome activation is probably key to understanding these differences and perhaps developing AMPs which are cancer-cell specific.

## Data Availability

The raw data supporting the conclusion of this article will be made available by the authors, without undue reservation.
