# Supplementary material for: Scorpion Venom Antimicrobial Peptides Induce Caspase-1 Dependant Pyroptotic Cell Death
Source: Front Pharmacol. 2022 Jan 10;12:788874. doi: 10.3389/fphar.2021.788874 (PMC8784870; doi:10.3389/fphar.2021.788874)
Supplement: Supplementary file 1 [file DataSheet1.docx]

| **Gene symbol** | **Gene Full name** | **Primer ID** | **Function** |
| --- | --- | --- | --- |
| **CASP8** | Caspase- 8  (cysteine-aspartic acid protease 8) | Hs01018151_m1 | Plays critical role in Apoptosis |
| **CASP 1** | Caspase -1  (cysteine-aspartic acid protease 1) | Hs00354836_m1 | Plays critical role in Pyroptosis |
| **MLKL** | mixed lineage kinase domain-like | Hs04188505_m1 | Plays critical role in Necroptosis |
| **NLRP3** | Nucleotide-binding domain and leucine-rich repeat protein-3 | Hs0091808_m1 | Plays critical role in Pyroptosis |
| **CAPN5** | Calpain 5  (calcium-dependant cystine proteases 5) | Hs00912756_m1 | Calcium dependant cell death  Necrosis |
| **GAPDH** | Glyceraldehyde-3-phosphate dehydrogenase | Hs99999905_m1 | Oxidoreductase in glycolysis and gluconeogenesis (Housekeeping gene) |

**Supplementary Data**

**Table S1.** qRT-PCR housekeeping and target gene information.


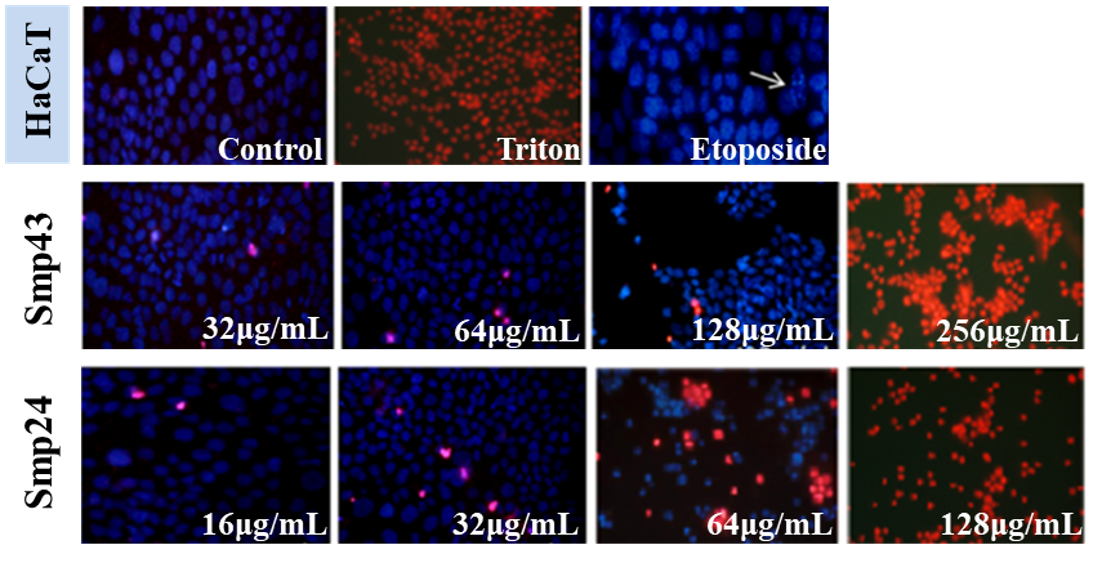


**Fig. S1.** Changes in nuclear morphology of the non-tumour (HaCaT) cell lines treated with different concentrations of Smp24 or Smp43 (8-256 μg/mL) showed significant increase in PI stain uptake in a concentration dependant manner (20X).


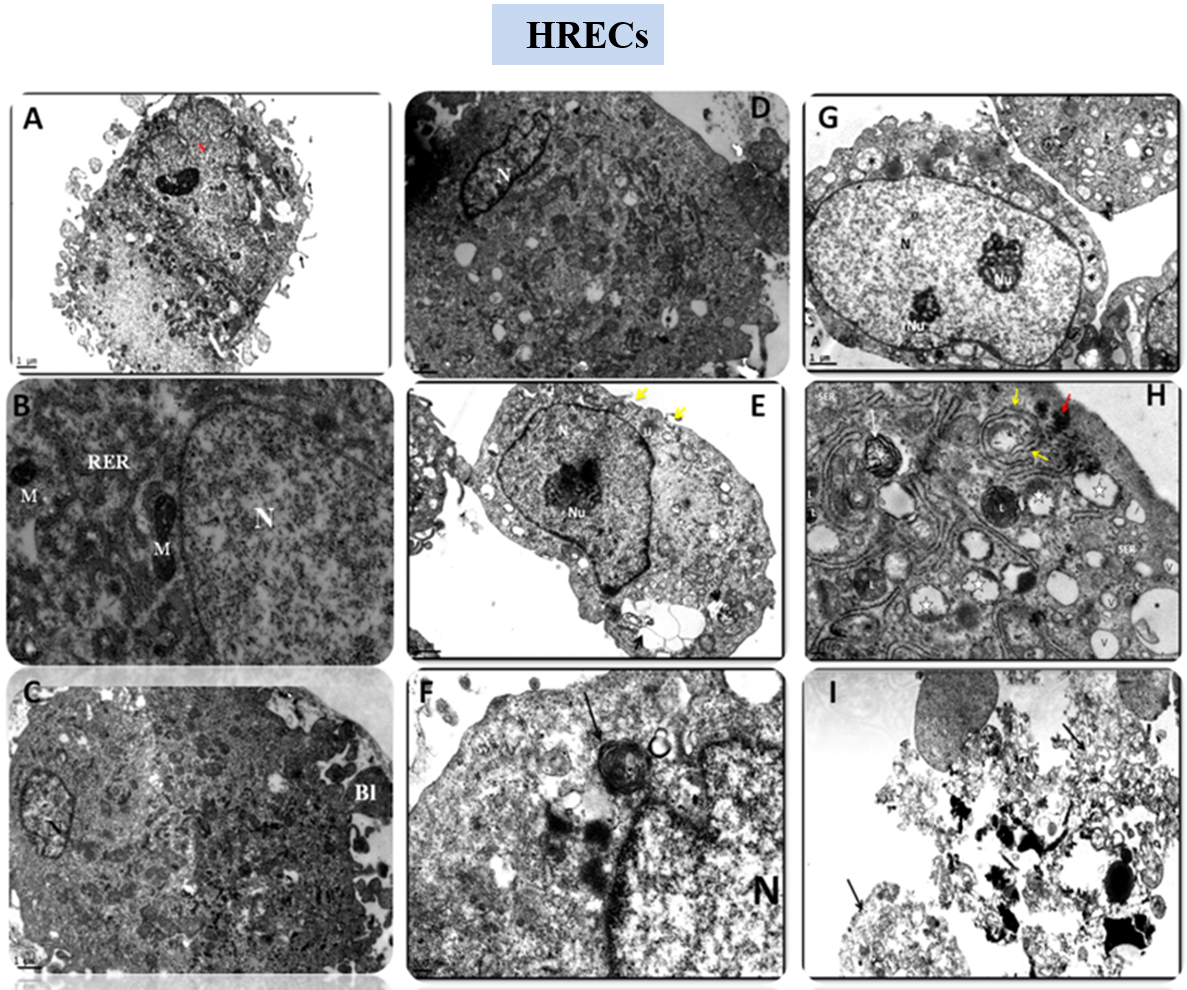


**Fig. S2.** Transmission electron micrograph of HRECs cells showing the effects of Smp24 and Smp43 treatment on their cell membrane. A, B control untreated cells showing normal ultrastructure of the cell. C and D cells treated with 1/2 LC_50_ concentration of Smp43 and Smp24, respectively, showing obvious increase in cell size and appearance of cell membrane blebs (Bl). E, F, G, H and I cells treated with LC_50_ concentration of Smp24 and Smp43. Most of cells were completely lysed (I) while other cells (E, G, H) showed ruptured cell membrane (yellow arrow), appearance of autolysosomes (black and yellow arrow) and many lucent vacuoles (star). Lamellar myelin structure (black and white arrow) were also observed (F, H).


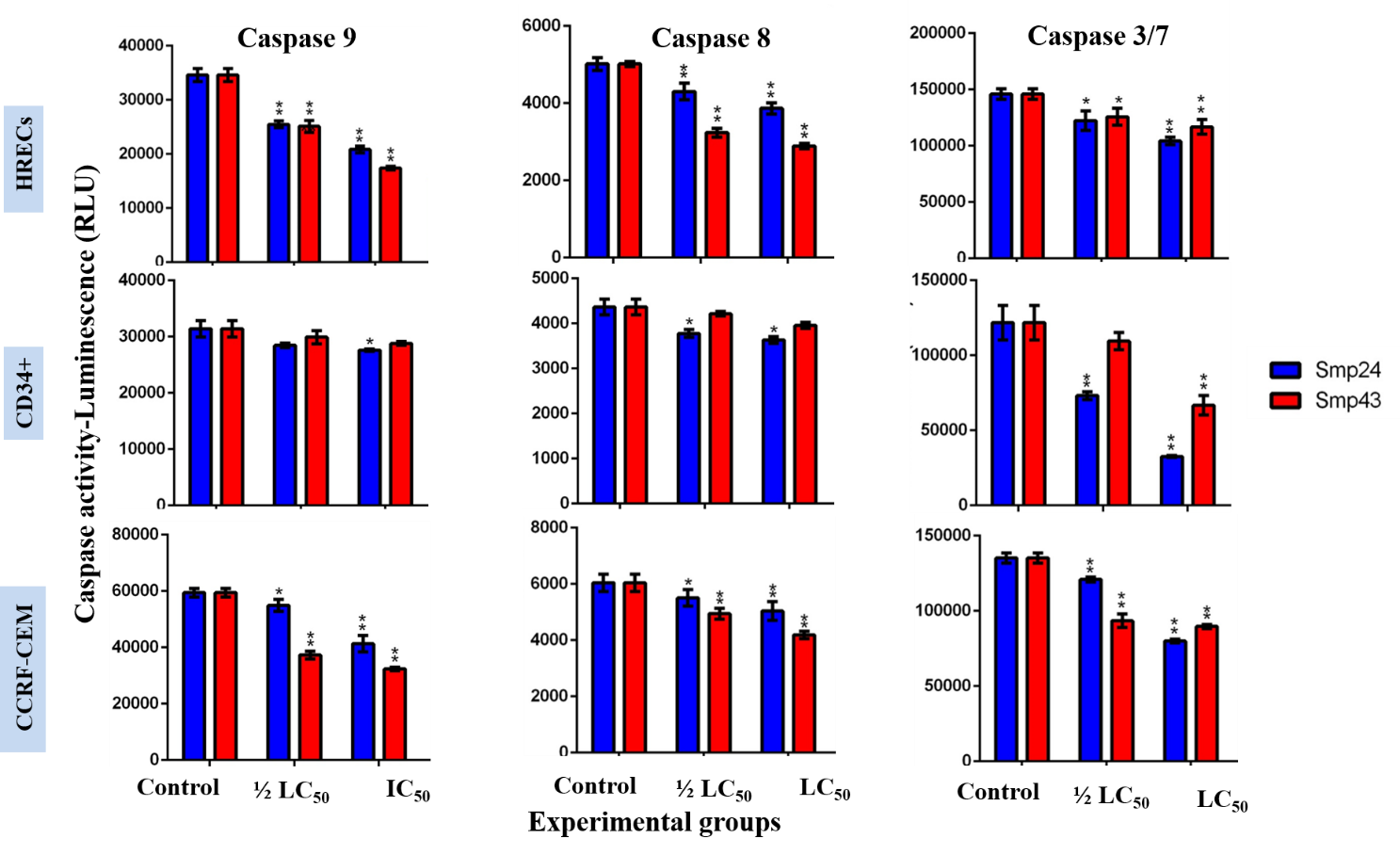


**Fig S3.** Charts showing the effect of Smp24 and Smp43 (1/2 LC_50_ and LC_50_) on the activity of caspase 8, 9 and 3/7 of representative non-tumour (HRECs and CD34+) and tumour (CCRF-CEM) cell lines. The results showed no significant increase in caspases activities. The data represented as mean ± SE. The statistical significance (* P ≤ 0.05, ** P ≤ 0.001) was determined by comparison with the control using student t-test.


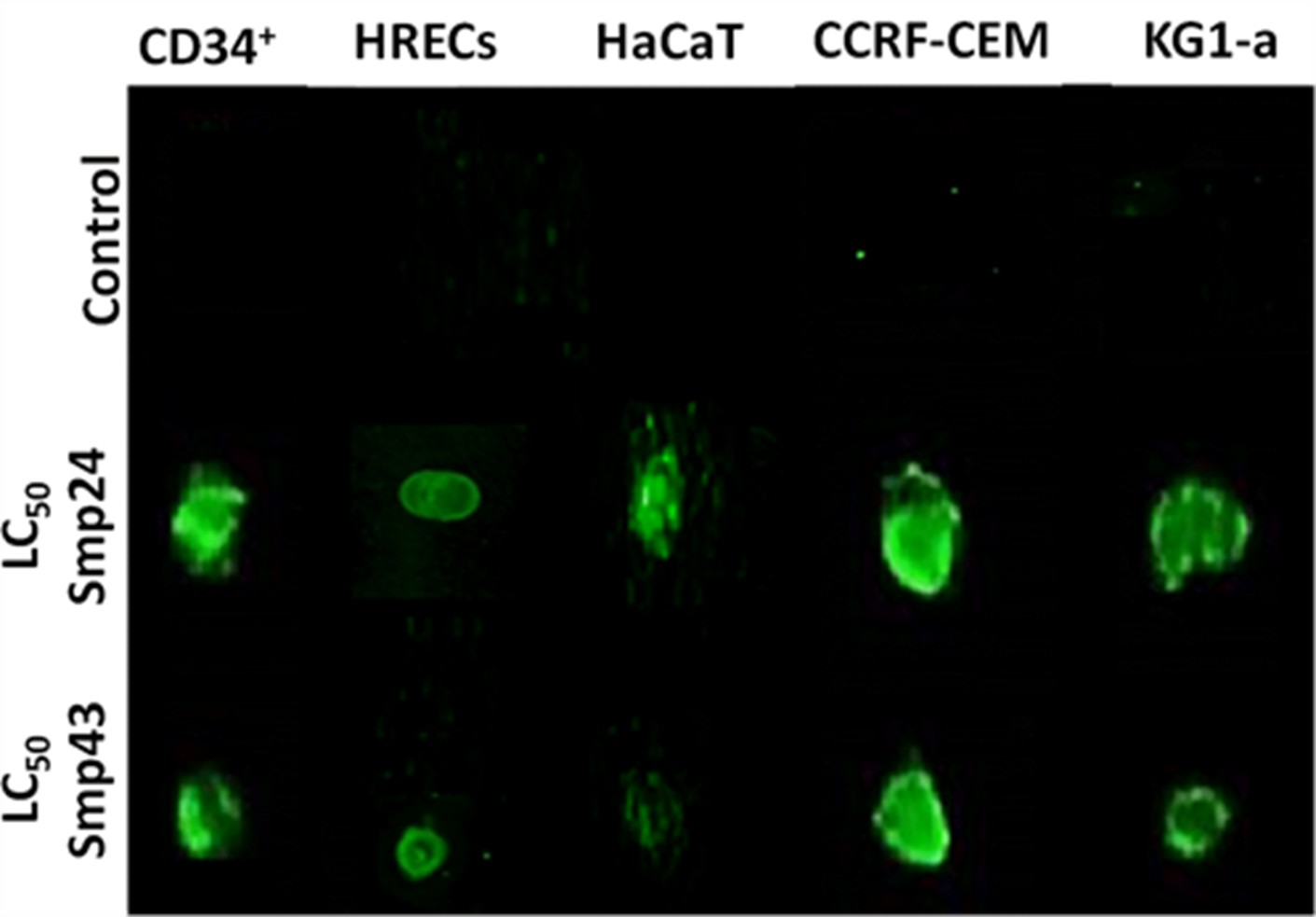


**Fig S4.** Dot blot analysis of culture supernatants of cells treated with Smp24 and Smp43 (LC_50_  concentrations) and immuonostained against Il-1B. The green dots represent samples where target IL-1B was present.
